# Adverse event profile of a mature voluntary medical male circumcision programme performing PrePex and surgical procedures in Zimbabwe

**DOI:** 10.7448/IAS.20.1.21394

**Published:** 2017-02-21

**Authors:** Aaron F Bochner, Caryl Feldacker, Batsi Makunike, Marrianne Holec, Vernon Murenje, Abby Stepaniak, Sinokuthemba Xaba, Shirish Balachandra, Mufuta Tshimanga, VTS Chitimbire, Scott Barnhart

**Affiliations:** ^a^ International Training and Education Center for Health (I-TECH), Seattle, WA, USA; ^b^ Department of Epidemiology, University of Washington, Seattle, WA, USA; ^c^ Department of Global Health, University of Washington, Seattle, WA, USA; ^d^ International Training and Education Center for Health (I-TECH), Harare, Zimbabwe; ^e^ Ministry of Health and Child Care, Harare, Zimbabwe; ^f^ U.S. Centers for Disease Control and Prevention, Harare, Zimbabwe; ^g^ Zimbabwe Community Health Intervention Project (ZiCHIRe), Harare, Zimbabwe; ^h^ Zimbabwe Association of Church-related Hospitals (ZACH), Harare, Zimbabwe; ^i^ Department of Medicine, University of Washington, Seattle, WA, USA

**Keywords:** Male circumcision, HIV prevention, adverse events, PrePex, Zimbabwe

## Abstract

**Introduction**: The frequency of adverse events (AEs) is a widely used indicator of voluntary medical male circumcision (VMMC) programme quality. Though over 11.7 million male circumcisions (MCs) have been performed, little published data exists on the profile of AEs from mature, large-scale programmes. No published data exists on routine implementation of PrePex, a device-based MC method.

**Methods**: The ZAZIC Consortium began implementing VMMC in Zimbabwe in 2013, supporting services at 36 facilities. Aggregate data on VMMC outputs are collected monthly from each facility. Detailed forms are completed describing the profile of each moderate and severe AE. Bivariate and multivariable analyses were conducted using log-binomial regression models.

**Results**: From October 2014 through September 2015, 44,868 clients were circumcised with 156 clients experiencing a moderate or severe AE. 96.2% of clients had a follow-up visit within 14 days of their procedure. AEs were uncommon, with 0.3% (116/41,416) of surgical and 1.2% (40/3,452) of PrePex clients experiencing a moderate or severe AE. After adjusting for VMMC site, we found that PrePex was associated with a 3.29-fold (95% CI: 1.78–6.06) increased risk of experiencing an AE compared to surgical procedures. Device displacements, when the PrePex device is intentionally or accidentally dislodged during the 7-day placement period, accounted for 70% of PrePex AEs. The majority of device displacements were intentional self-removals. Overall, infection was the most common AE among VMMC clients. Compared to clients aged 20 and above, clients aged 10–14 were 3.07-fold (95% CI: 1.36–6.91) more likely to experience an infection and clients aged 15–19 were 1.80-fold (95% CI: 0.82–3.92) more likely to experience an infection, adjusted for site.

**Conclusions**: This exploratory analysis found that clients receiving PrePex were more likely to experience an AE than surgical circumcision clients. This is largely attributable to the occurrence of device displacements, which require prompt access to corrective surgical MC procedures as part of their clinical management. Most device displacements were self-removals which are preventable if client behaviour could be modified through counselling interventions. We also found that infection after MC is more common among younger clients, who may benefit from additional counselling or increased parental involvement.

## Introduction

After studies found that male circumcision (MC) reduces the risk of female-to-male HIV-1 transmission by up to 60% [1–3], the World Health Organization (WHO) and the US President’s Emergency Plan for AIDS Relief (PEPFAR) began supporting implementation of voluntary medical male circumcision (VMMC) programmes across 14 priority African countries. From 2008 through 2015, nearly 11.7 million VMMC procedures were performed. However, this represents only 56% of the target of 20.8 million procedures needed to achieve 80% coverage in these priority countries, so scale up of high-quality VMMC programmes must continue for VMMC to have its intended impact on the HIV epidemic [4].

The proportion of clients experiencing adverse events (AEs) from MC is a widely used indicator of VMMC programme quality. The frequency of moderate and severe AEs from controlled trials and pilot programmes have ranged from 0.5% to 8% [1–3,5–15]. Because these studies generally include highly trained providers, frequent follow-up visits, and high follow-up rates, these findings may not be generalizable to routine programme implementation. Additionally, these studies have had limited statistical power to detect factors associated with the risk of experiencing adverse events [5,12]. To date, there is limited published data on the profile of AEs within mature, large-scale VMMC programmes [16–18], and no data on routine implementation of PrePex, a device-based MC method that received WHO prequalification in May 2013 [19].

As scale-up of VMMC advances, the ability to provide high-quality VMMC services to clients of large VMMC programmes is essential for both client safety and programmatic success. We present the profile of AEs that occurred over one year of VMMC programme implementation in Zimbabwe, where both surgical and PrePex MCs were offered. Additionally, we performed an exploratory analysis of factors associated with the risk of experiencing an AE. Results from our analysis may help other VMMC programmes evaluate their AE profile and create focused counselling messages to reduce the occurrence of AEs.

## Methods

### VMMC programme

The ZAZIC consortium has supported VMMC services in 21 districts across all 10 provinces in Zimbabwe. ZAZIC is comprised of four organizations: The International Training and Education Center for Health (I-TECH), the University of Zimbabwe-University of California San Francisco Collaborative Research Program (UZ-UCSF), the Zimbabwe Association of Church-related Hospitals (ZACH), and the Zimbabwe Community Health Intervention Research Project (ZiCHIRe). The programme began implementation in 2013 in collaboration with the Zimbabwe Ministry of Health and Child Care (MOHCC) and is supported by a cooperative agreement with the U.S. Centers for Disease Control and Prevention (CDC). Prior to October 2014, the programme had performed 46,011 procedures.

### Study population

From October 2014 through September 2015, ZAZIC supported VMMC services at 36 sites in Zimbabwe. These facilities were primarily hospitals, and trained staff offered VMMC services at the facility where they were based (hereafter referred to as static facilities), at outreach facilities (often rural health centres or workplaces), or using mobile caravans. The programme performed VMMC on clients aged 10 and above.

Three circumcision techniques were used at ZAZIC facilities. The forceps-guided method had been the standard MC technique used in Zimbabwe. In April 2014, PrePex was approved for clients aged 18 and above, so adult clients had access to PrePex and surgical MC procedures. In August 2014, due to safety concerns, PEPFAR and MOHCC prohibited the use of forceps-guided MC among clients under age 15 and the dorsal slit technique was introduced. Adult clients were circumcised by the MC method of their choice, though availability of PrePex and surgical procedures varied by site and over time as staff received training in different MC methods. At the start of the study period, three ZAZIC sites were offering PrePex procedures, but PrePex services expanded to a total of 19 sites by September 2015.

The information included in this report are routinely collected programmatic data and do not constitute human subjects research. The Medical Research Council of Zimbabwe, the CDC, and the University of Washington’s Internal Review Board provided non-research determination for this routine programme implementation analysis.

### Data collection and statistical analysis

This analysis utilized three data sources. An MOHCC Monthly Return Form was completed by sites at the end of each month, reporting monthly VMMC statistics disaggregated by age category, HIV-1 status, VMMC method, the number of moderate or severe AEs, and if a follow-up visit occurred within 14 days of the circumcision procedure. VMMC method was not requested on the form, but sites reported disaggregation by VMMC method in the comments section. ZAZIC communicates weekly with sites to obtain the number of MCs done in the prior week and also collects information on the location type where MCs were performed (static, outreach, or caravan). Since totals collected from these weekly communications did not always match the official totals reported on Monthly Return Forms, information on the location type was scaled for each site and assumed to be missing at random. Details on adverse events are collected using the ZAZIC Adverse Event Review Tool, a ZAZIC-developed form that collects detailed information on moderate and severe AEs, including the history, examination findings, management, and clinical outcomes.

Adverse events were categorized according to standard PEPFAR definitions [20]. Among clients who experienced more than one category of AE, only the most severe AE was reported. Bivariate and multivariable analyses were conducted using log-binomial regression models with robust standard errors, with multivariable analyses allowing for clustering by site. Analyses were conducted using Stata version 12.1 (Stata Corporation, College Station, TX).

## Results and discussion

ZAZIC’s VMMC programme circumcised a total of 44,868 clients from October 2014 through September 2015. The volume of MCs performed across the 36 sites varied greatly, ranging from 202 to 5,919 procedures. The majority of clients were school-age, most procedures were done at outreach or mobile facilities, and 92.3% of clients received a surgical circumcision (Table 1). The programme also had high follow-up rates, with 96.2% of clients having at least one follow-up visit within 14 days of their MC.

**Table 1.** Characteristics of VMMC clients from October 2014 to September 2015

Characteristics	VMMC Clients (*N* = 44,868) n (%)
Age	
10–14	19,619 (43.7%)
15–19	15,129 (33.7%)
20–24	4,816 (10.7%)
25–29	2,508 (5.6%)
30–49	2,541 (5.7%)
>50	255 (0.6%)
MC method	
Surgical (forceps-guided or dorsal slit)	41,416 (92.3%)
PrePex	3,452 (7.7%)
HIV-1 serostatus	
Negative	44,484 (99.2%)
Positive	230 (0.5%)
Unknown	154 (0.3%)
Location type where MC was performed	
Static facility	10,459 (23.3%)
Outreach or caravan	34,409 (76.7%)
Follow-up visit within 14 days of MC	
Yes	43,180 (96.2%)
No	1,688 (3.8%)

Among VMMC clients, 0.3% (156/44,868) experienced a moderate or severe AE. Infection was the most common type of AE, followed by device displacement and bleeding (Table 2). Of the 156 AEs, 116 occurred among clients who received a surgical procedure. Only six of the surgical AEs occurred intraoperatively, while 110 occurred postoperatively. Forty AEs occurred among clients with PrePex. As expected, all 40 clients who received PrePex and experienced an AE were aged 18 and above. Device displacements, when the PrePex device is intentionally or accidentally dislodged during the 7-day placement period, accounted for 70% of PrePex AEs. Device displacements, the most common severe AE, were categorized as severe if a surgical MC procedure was required for clinical management. According to client self-report, 19 of the 28 device displacements were caused by the client intentionally removing the device: seven clients removed the device due to pain, four removed the device to have sex, three removed the device for other reasons, and five clients did not report a reason for removing the device. Among the nine clients who accidently dislodged the device, four clients reported that the device moved while they were having sex, three did not know what caused the device to move, one client accidently displaced the device while scratching around the area, and one client had it move while playing soccer.

**Table 2.** Moderate and severe adverse events listed by male circumcision method

	Intraoperative		Postoperative			
AE type	Moderate	Severe	Moderate	Severe	Totals	Rate per 10,000 MCs
Surgical						
Bleeding	2	1	13	4	20	4.8
Infection	0	0	57	10	67	16.2
Insufficient skin removal	0	0	0	3	3	0.7
Other	0	0	0	1	1	0.2
Pain	0	1	0	0	1	0.2
Scarring, disfigurement, or damage to penis	0	2	0	1	3	0.7
Swelling	0	0	2	3	5	1.2
Wound disruption	0	0	14	2	16	3.9
Subtotal	2	4	86	24	116	28.0
PrePex						
Device displacement	-	-	2	26	28	81.1
Infection	-	-	7	0	7	20.3
Pain	-	-	3	1	4	11.6
Swelling	-	-	1	0	1	2.9
Subtotal	-	-	13	27	40	115.9
Total AEs	2	4	99	51	156	34.8

Using aggregate data, we explored if age, VMMC method, or location type were associated with the risk of experiencing an AE (Table 3). After adjusting for the 36 VMMC sites, we found that PrePex was associated with a 3.29-fold (95% CI: 1.78–6.06) increased risk of experiencing a moderate or severe AE. Overall, AEs were uncommon among clients receiving either MC method, with 0.28% of surgical and 1.16% of PrePex clients experiencing an AE. In the unadjusted analysis a higher proportion of clients at static facilities experienced an AE compared to clients attending outreach facilities or caravans (RR = 1.61, 95% CI: 1.14–2.27), but this was no longer statistically significant after adjusting for site. We did not detect an association between age and the risk of experiencing an AE.

**Table 3.** Factors associated with experiencing a moderate or severe adverse event

	N with AE/Total (%)	Unadjusted RR (95% CI)	Adjusted RR (95% CI)^1^
Age			
≥20	43/10,120 (0.42%)	1.00 (ref)	1.00 (ref)
15–19	49/15,129 (0.32%)	0.76 (0.51–1.15)	0.87 (0.56–1.36)
10–14	64/19,619 (0.33%)	0.77 (0.52–1.13)	0.83 (0.49–1.39)
MC method			
Surgical (forceps-guided or dorsal slit)	116/41,416 (0.28%)	1.00 (ref)	1.00 (ref)
PrePex	40/3,452 (1.16%)	4.14 (2.89–5.92)	3.29 (1.78–6.06)^2^
Location type where MC was performed^3^			
Outreach or caravan	98/34,409 (0.29%)	1.00 (ref)	1.00 (ref)
Static facility	48/10,459 (0.46%)	1.61 (1.14–2.27)	1.14 (0.56–2.30)

^1^ Adjusted for site and allowing for clustering. Eight sites were excluded from the adjusted analysis due to having no adverse events. An additional nine sites were excluded from the adjusted MC method analysis because those sites only conducted surgical procedures. N = 36,534 MC clients included in the age and location adjusted analysis, N = 23,777 MC clients included in the MC method analysis.

^2^ P < 0.0005

^3^ For 10 AEs, the location where the MC was performed was not recorded.

Since infection was the most common AE among VMMC clients overall, we assessed factors associated with increased risk of infection (Table 4). We found that compared to clients aged 20 and above, clients aged 10–14 were 3.07-fold (95% CI: 1.36–6.91) more likely to experience an infection and clients aged 15–19 were 1.80-fold (95% CI: 0.82–3.92) more likely to experience an infection. MC method and location type were not associated with statistically significant differences in the risk of experiencing a moderate or severe infection.

**Table 4.** Factors associated with experiencing moderate or severe infection

	N with Inf./Total (%)	Unadjusted RR (95% CI)	Adjusted RR (95% CI)^1^
Age			
≥20	6/10,120 (0.06%)	1.00 (ref)	1.00 (ref)
15–19	21/15,129 (0.14%)	2.34 (0.95–5.80)	1.80 (0.82–3.92)
10–14	47/19,619 (0.24%)	4.04 (1.73–9.45)	3.07 (1.36–6.91)^2^
MC method			
Surgical (forceps-guided or dorsal slit)	67/41,416 (0.16%)	1.00 (ref)	1.00 (ref)
PrePex	7/3,452 (0.20%)	1.25 (0.58–2.73)	1.43 (0.52–3.93)
Location type where MC was performed^3^			
Outreach or caravan	56/34,409 (0.16%)	1.00 (ref)	1.00 (ref)
Static facility	16/10,459 (0.15%)	0.94 (0.54–1.64)	1.21 (0.41–3.58)

^1^ Analyses adjusted for site and allowing for clustering. 18 sites were excluded from the adjusted analysis due to having no infection adverse events. N = 24,370 MC clients included in the analysis.

^2^ P < 0.01

^3^ For two infection AEs, the location where the MC was performed was not recorded.

To the best of our knowledge, this exploratory analysis is the first to find that clients who received an MC using PrePex were more likely to experience an AE than those receiving surgical circumcision. Among those with a PrePex MC, 0.8% of clients experienced a device displacement, similar to findings from other studies [9–15]. Excluding device displacement, the proportion of PrePex clients who had an AE (0.35%) was similar to surgical clients (0.28%). All device displacements were successfully managed, and none of these clients required hospitalization or experienced permanent deformity or disability. One advantage of PrePex is that it can be performed by lower cadres of healthcare workers compared to surgical MC [19], and this has led to PrePex services expanding to facilities without on-site staff trained to perform surgical MC. However, the regular occurrence of device displacements is an important consideration because device displacements almost always require a surgical MC procedure as a component of AE management. To avoid complications, including oedema, urinary tract obstruction, and potential tissue devitalisation, programmes must have mechanisms in place to promptly provide these clients with surgical MC procedures. We found that most device displacements occurred when the client intentionally removed the device or engaged in sexual intercourse, suggesting that improved education and counselling have the potential to reduce the frequency of device displacements.

Our analysis also found that infection was the most common type of AE following a surgical VMMC procedure, consistent with findings from other large-scale VMMC programmes [16–18]. We are the first to assess risk factors for infection after VMMC. Our finding that clients aged 10–14 had a 3-fold higher risk of infection compared to clients aged 20 and above, but no elevated risk of AEs overall, suggests that younger clients may be less likely to perform recommended wound care. New approaches for counselling young clients on wound care or increased parental engagement may be beneficial in reducing the proportion of young clients who experience an infection. Though not statistically significant, our analysis suggests that clients aged 15–19 may also be at higher risk of infection compared to older clients. We found no evidence that infection risk increased among clients circumcised at outreach sites, which is important as communities living near static sites reach saturation and an increasing proportion of MCs are done at outreach locations.

This analysis had several important limitations. Though we were able to adjust across our 36 sites, it was not possible to adjust for other factors in multivariate analyses because we relied on aggregate data sources. Thus, there may be confounding that biases our results. Additionally, the low proportion of clients with AEs compared to findings from controlled trials suggests that AEs may have been underreported. We rely on a passive surveillance system for AE reporting, and a previous study showed such systems underreport AEs [16]. Though adjusting for site controls for reporting difference across sites, it is possible that some types of AEs were more or less likely to be reported. For example, sites may be more likely to report AEs such as infections or device displacements that were caused by client behaviour compared to AEs caused by clinician error. Last, when adjusting for AE method, we could not analyse forceps-guided and dorsal slit procedures separately because our reporting system only collected the total number of surgical procedures.

Strengths of this analysis include the large sample size and number of AEs, which provide the statistical power to detect weaker associations. The VMMC programme has a high client follow-up rate, allowing clinicians the opportunity to detect and report the occurrence of AEs. Additionally, information captured on the ZAZIC Adverse Event Review Tool provided a large amount of high-quality data on each AE. Last, the observed association between MC method and AEs as well as the observed associations between age and risk of infection were both strong associations with very plausible explanations, making it less likely that the associations occurred due to confounding or chance.

## Conclusions

Considering the large number of VMMCs that have been performed over the past few years, a surprising paucity of programmatic data has been published to-date. With VMMC programmes roughly 50% of the way to achieving targets set by the WHO and PEPFAR, large-scale VMMC programmes will continue for several years to come. Our finding that device displacements accounted for a majority of PrePex AEs highlights the need for VMMC programmes to have mechanisms in place to ensure that all PrePex clients have prompt access to corrective surgical MC procedures as a component of AE management. The fact that the majority of device displacements were potentially preventable self-removals and that younger clients were at greater risk of infection can help VMMC programmes improve counselling messages and programme quality.

## Abbreviations

AE: adverse event; CDC: U.S. Centers for Disease Control and Prevention; I-TECH: International Training and Education Center for Health; MC: male circumcision; MOHCC: Ministry of Health and Child Care; PEPFAR: The U.S. President’s Emergency Plan For AIDS Relief; UZ-UCSF: University of Zimbabwe-University of California San Francisco Collaborative Research Program; VMMC: voluntary medical male circumcision; WHO: World Health Organization; ZACH: Zimbabwe Association of Church-related Hospitals; ZiCHIRe: Zimbabwe Community Health Intervention Research Project.
